# Effects of a Clinically Indicated Peripheral Intravenous Replacement on Indwelling Time and Complications of Peripheral Intravenous Catheters in Pediatric Patients: A Randomized Controlled Trial

**DOI:** 10.3390/ijerph18073795

**Published:** 2021-04-05

**Authors:** Su-Wen Lin, Shu-Ching Chen, Fang-Yi Huang, Ming-Ying Lee, Chun-Chu Chang

**Affiliations:** 1 Department of Nursing, Chang Gung Memorial Hospital at Linkou, Taoyuan 333, Taiwan; a2213@cgmh.org.tw (S.-W.L.); iren@cgmh.org.tw (M.-Y.L.); 2 School of Nursing and Geriatric and Long-Term Care Research Center, College of Nursing, Chang Gung University of Science and Technology, Taoyuan 333, Taiwan; shuching@gw.cgust.edu.tw; 3 Department of Radiation Oncology and Proton and Radiation Therapy Center, Chang Gung Memorial Hospital at Linkou, Taoyuan 333, Taiwan; 4 School of Nursing, College of Medicine, Chang Gung University, Taoyuan 333, Taiwan; 5 Department of Nursing, New Taipei Municipal Tucheng Hospital Chang Gung Memorial Hospital, New Taipei 236, Taiwan; fy1233@adm.cgmh.org.tw; 6 Department of Nursing, College of Nursing, Chang Gung University of Science and Technology, Taoyuan 333, Taiwan

**Keywords:** pediatric patients, peripheral intravenous catheters, clinically indicated peripheral intravenous replacement, indwelling time, complications

## Abstract

Peripheral intravenous catheters (PVCs) are common treatment modalities for pediatric patients, and may cause infection, infiltration, occlusion, and phlebitis. The purpose of this study was to evaluate the effect of a clinically indicated peripheral intravenous replacement (CIPIR) on PVC indwelling time and complication rates in pediatric patients. This study used a randomized, pre- and post-repeated measures design. A total of 283 participants were randomly assigned to an experimental group (*n* = 140) and a control group (*n* = 143). The experimental group received CIPIR and the control group received usual care with routine PVC replacement every three days. The insert sites of PVC were assessed every day until the signs of infiltration, occlusion, or phlebitis were presented. Patients in the experimental group had significantly longer PVC indwelling times compared to those in the control group (*t* = −18.447, *p* < 0.001). No significant differences were noted between groups in infiltration (χ^2^ = 2.193, *p* = 0.139), occlusion (χ^2^ = 0.498, *p* = 0.481), or phlebitis (χ^2^ = 3.865, *p* = 0.050). CIPIR can prolong the PVC indwelling time in pediatric patients with no increase in the rate of adverse events.

## 1. Introduction

Peripheral intravenous catheters (PVCs) are common treatment modalities [1,2]. More than 80% of hospitalized patients receive PVCs [3], which provide medication, blood transfusion, fluid infusion, and nutritional supplements [1,4]. Pediatric patients with fever, infection, gastroenteritis, or other conditions often receive PVCs for blood transfusion, medication, or resuscitation [5]. PVCs may cause complications such as infection, infiltration, occlusion, or phlebitis [1,4]. Approximately 28.0% of patients with PVCs have complications, and pediatric patients have a higher prevalence of such complications than adults [1]. Phlebitis, for example, is associated with a longer PVC indwelling time [6].

## 2. Literature Review

Based on recommendations from the Centers for Disease Control and Prevention (CDC) and the Healthcare Infection Control Practices Advisory Committee (HICPAC), clinical practice guidelines for preventing intravascular catheter-related infections recommend avoiding the routine replacement of PVCs as a strategy to prevent infection [6]. A study of pediatric intensive care unit patients by Garland et al. [7] revealed that replacing catheters in critically ill children every 72 h did not decrease the incidence of phlebitis, bacterial colonization, or catheter-induced sepsis, and could increase extravasation risk. In critically ill children, colonization risk increased from 11% in catheters that were in situ for 48 to 144 h, to 34% for catheters that were in for longer than 144 h. Catheters maintained up to 144 h in critically ill children should be monitored. Factors associated with extravasation include phlebitis correlated with infusion of hyperalimentation or lorazepam, catheter location, younger age, catheter time in situ ≤72 h, and the infusion of antiepileptics. Oishi et al. reviewed the relevant literature [8] and found that changing catheters every 48–72 h reduced the risk of complications such as phlebitis and infection. Shimandle et al. [9] reported that neither the per-day risk of phlebitis nor that of catheter colonization increased significantly with placement >3 days. They also found that the overall risk of PVC-related complications in children is extremely low, and routine catheter replacement did not reduce it substantially. Foster et al. [10] indicated that the average indwelling time of PVCs was 42.35 h (range = 2.5–189.5 h), 13% of pediatric patients had PVC indwelling times of >72 h, and 5.7% of pediatric patients had PVC indwelling times of >96 h. A recent study [11] indicated that the incidence of infiltration, occlusion, and phlebitis of PVC were 17.8%, 10.8%, and 10.5% among 1069 adult patients, respectively, most complications in phlebitis (88.4%) and infiltration (93.7%) were grade 1. The study also showed that the clinically indicating instead the routine replacement of catheters is safe [11]. A cohort study of pediatric patients with arterial catheterization by King et al. [12] revealed that 10.3% of patients reported complications, and younger age; insertion of catheters after the first day in hospital; and the need for cardiac surgery, bone marrow transplantation, and dialysis were associated with complication of arterial catheterization. Cicolini et al. [13] found that the average time for catheters in situ was 65.6 h and 23.6% of the catheters were in place beyond 96 h, phlebitis incidence was 15.4%, 94.4% of which were grade 1, and catheter duration after 96 h being most highly associated with phlebitis. A study by Bregenzer et al. [14] followed 609 catheters for 1 to 28 days, which indicated that phlebitis, catheter-related infection, and obstruction occurred in 19.7%, 6.9%, and 6.0% of catheters, respectively, and phlebitis, catheter-related infections, and mechanical complications did not increase during prolonged catheterization. Based on the aforementioned studies, we hypothesized that a clinically indicated peripheral intravenous replacement (CIPIR) can decrease the routine replacement of PVC and alleviate discomfort to the patients, prolonging the PVC indwelling time but not significantly affecting the rates of complications of infiltration, occlusion, and phlebitis; and that routine catheter replacement is not necessary. Therefore, the purpose of this study was to evaluate the effects of CIPIR on PVC indwelling time and the rates of complications of PVCs in pediatric patients.

## 3. Materials and Methods

### 3.1. Design and Population

#### 3.1.1. Design

This study was a randomized controlled trial, an unblinded study using a pre- and post-repeated measures design. The study was conducted between September 2016 and February 2019. Participants in the study were randomly assigned to the experimental group or control group using computer-generated random numbers. The experimental group received a CIPIR based on guidelines for the prevention of intravascular catheter-related infections, while the control group received usual care.

#### 3.1.2. Participant Sample and Setting

A convenience sample of pediatric patients was selected from the pediatric wards of a medical center in northern Taiwan. Pediatric patients who met the inclusion criteria were (1) diagnosed with an infection-related disease, including pneumonia, urinary tract infection, upper respiratory infection, or gastroenteritis; and (2) had a treatment plan that included a PVC. Pediatric patients were not eligible if they had an unstable systemic disease (valvular heart disease, sepsis, or other underlying disease), previous peripheral vascular diseases, or used immunosuppressive drugs or combined central intravenous catheters (all based on medical records). The inclusion criteria for family caregivers were (1) identification as the primary family caregiver of the pediatric patient, providing uncompensated care or assistance; (2) older than 20 years of age; (3) was advised by the attending physician of the treatment plan and awaiting a PVC; and (4) Mandarin or Taiwanese speaking. Potential subjects were recruited from referrals by the primary registered nurse.

#### 3.1.3. Sample Size Calculation

The power of this study was estimated based on PVC dwell time. Based on a previous similar study, an effect size of 1.15 was required to detect differences between the two groups [15]. A sample size of 140 per group was found sufficient to detect an effect size of 1.15, with significance set at an α of 0.05, a power of 0.8, and considering the clinical situation’s impact on the participants’ attrition, such as transfer to other hospitals, a change in treatment plan, primary caregivers asking to change the site of the PVC, using an estimated 30% attrition rate to detect statistically significant differences between the two study groups [16].

#### 3.1.4. Ethical Consideration

The study was approved by the Ethical Review Board of the study institute (Number: 103–1023B). All participants (via the family caregiver) provided written informed consent to participate in the study before any data were collected.

#### 3.1.5. Intervention Program and Data Collection Process

The control group received usual care, which was the routine replacement of the PVC every three days. The experimental group received CIPIR, which was the replacement of PVC until infiltration, occlusion, or phlebitis.

The time points of data collection were at the initial receiving of a PVC (pre-test), and then per-day following the receiving of the PVC, and the endpoints were infiltration, occlusion, or phlebitis. The insert sites of PVC were assessed hourly of every day until the signs of infiltration, occlusion, or phlebitis were presented [11,15,17].

### 3.2. Outcome Measures

#### 3.2.1. Pediatric Patients

##### The Infusion Nurses Society Infiltration Scale (INSIS)

The Infusion Nurses Society infiltration scale (INSIS) was used to assess the infiltration of PVCs. The grade of infiltration is measured in five grades, ranging from 0 to 4: grade 0, no symptoms; grade 1, skin blanched, edema < 1 in (2.5 cm) in any direction, cool to the touch with or without pain; grade 2, skin blanched, edema 1 to 6 in (2.5–15 cm) in any direction, cool to the touch with or without pain; grade 3, skin blanched, translucent gross edema 6 in (15 cm) in any direction, cool to the touch with mild to moderate pain and possible numbness; grade 4, skin blanched, translucent skin, leaking skin discolored or bruised, swollen gross edema > 6 in (15 cm) in any direction, deep pitting tissue edema, circulatory impairment, moderate to severe pain, infiltration of any amount of blood product, irritant, or vesicant [18]. The INSIS has been widely used and demonstrated to be reliable in a peripheral vascular-related study [19]. The inter-rater reliability for this study was 0.99 between the research nurses and the principal investigator, who provided training to the research nurses.

##### The Occlusion Scale (OS)

We developed the occlusion scale (OS) based on literature review [19,20] to assess the occlusion of PVCs, including blood clots, medication sedimentation, medication flocculation, medication crystallate, and blood backflow. The presence of any of the above problems was scored as 1, and none of the above problems was scored as 0. The OS was checked by five infection and pediatric experts (one pediatric physician, two pediatric nurses, and two infection control nurses) and the content validity index (CVI) was 0.97. The inter-rater reliability for this study was 0.98 between the research nurses and the principal investigator, who provided training to the research nurses. The scale was pilot-tested on five pediatric patients and demonstrated satisfactory psychometric properties.

##### The Infusion Nurses Society Phlebitis Scale (INSPS)

The Infusion Nurses Society phlebitis scale (INSPS) was used to assess phlebitis in PVCs. The scale consists of five grades: grade 0, no symptoms; grade 1, erythema at the access site, with or without pain; grade 2, pain at the access site with erythema and/or edema; grade 3, pain at the access site with erythema and/or edema, streak formation, and palpable venous cord; and grade 4, pain at the access site with erythema and/or edema, streak formation; palpable venous cord > 1 in (2.5 cm) in length, and purulent drainage [19]. A previous study showed satisfactory psychometric properties for the INSPS [19]. The inter-rater reliability for this study was 0.98 between the research nurses and the principal investigator, who provided training to the research nurses.

##### Demographic and Clinical Characteristics

Demographic data collected included patient age and gender. Disease and treatment characteristics included diagnosis, PVC site (right forearm or hand, left forearm or hand, right lower extremity, or left lower extremity), and hospital length of stay (LOS).

#### 3.2.2. Family Caregivers

##### Demographic and Care Characteristics of Family Caregivers

The data collected from the family caregivers were age, occupation, educational level, and relationship to the pediatric patient.

### 3.3. Statistical Methods

Data were analyzed using SPSS, version 26.0 for Windows (IBM Corp., Armonk, NY, USA). Descriptive statistics were used to summarize the demographic and clinical characteristics of the pediatric patients and the family caregivers. A χ^2^ test, Fisher’s exact test, and an independent t-test were used to test the homogeneity between the two groups at baseline, as well as differences in age, gender, diagnosis, PVC site, and hospitalization LOS. The effects of CIPIR on PVC indwelling time and rates of complications (infiltration, occlusion, and phlebitis) were analyzed using a χ^2^ test, Fisher’s exact test, and an independent t-test, as appropriate. Relative risk, number needed to treat, relative risk reduction, and absolute risk reduction were analyzed for the CIPIR on rates of complications (infiltration, occlusion, and phlebitis) between the two groups. Two-tailed t-tests were used for comparisons, and a value of *p* < 0.05 represented statistical significance.

## 4. Results

### 4.1. Study Participant Flow

**Figure 1:**
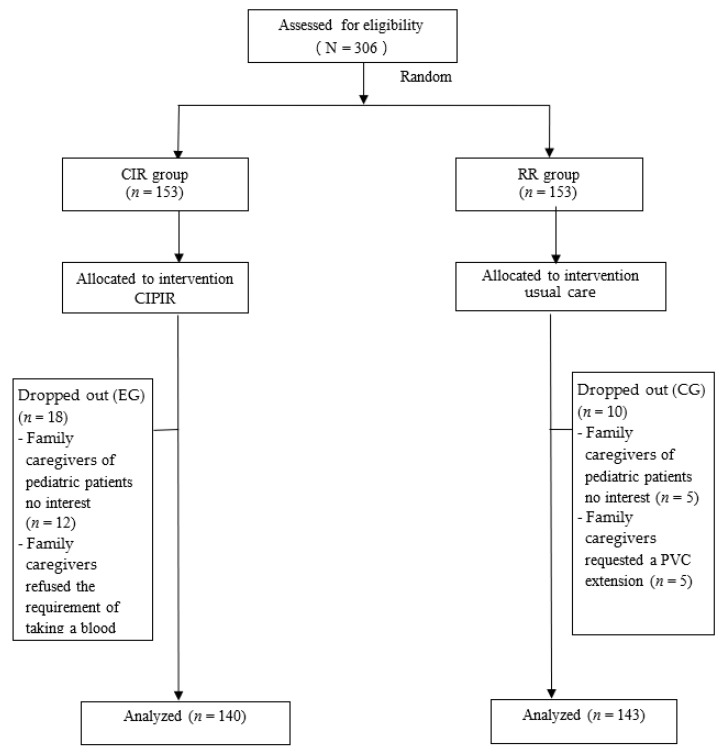
Study participant flow.

Of 306 eligible pediatric patient–family caregivers dyads recruited, in the experimental group, 5 parents of pediatric patients declined to participate because of no interest, 7 family caregivers were grandparents who declined to participate because of no interest, and 6 family caregivers refused the requirement of taking a blood sample to determine the disease condition. In the control group, 5 family caregivers of pediatric patients declined to participate because of no interest and 5 family caregivers requested a PVC extension, which made the subject ineligible for further study (Figure 1).

As a result, 283 pediatric patient–family caregiver dyads who completed all assessments were included in the analysis, 140 in the experimental group and 143 in the control group.

### 4.2. Pediatric Patients and Disease Characteristics

Patients in the experimental group and control group were, on average, 3.44 (standard error of the mean, SE = 0.34) and 4.42 (SE = 0.36) years old, respectively. The majority of patients in the control group were male (56.6%). In both groups, the most common diagnosis was respiratory system disease. The majority of participants had PVCs in the left forearm or hand. There were no statistically significant differences between the two groups in terms of patient or disease characteristics. Patient and disease characteristics are summarized in Table 1.

### 4.3. Indwelling Time and Rates of Complications Associated with Peripheral Intravenous Catheters (PVCs) at Post-Test between the Two Groups

Patients in the experimental group (mean = 113.06 h, SE = 2.42) had a longer PVC indwelling time than patients in the control group (mean = 65.67 h, SE = 0.88), with statistically significant differences (t = −18.447, *p* < 0.01). No statistically significant differences were noted between groups in terms of the rates of infiltration, occlusion, or phlebitis (Table 2).

### 4.4. Relative Risk, Number Needed to Treat, Relative Risk Reduction, and Absolute Risk Reduction between the Two Groups

The results of relative risk (95% confidence interval), number needed to treat, relative risk reduction, and absolute risk reduction for differences of the rates of infiltration, occlusion, and phlebitis, in the experimental group and the control group, respectively, are shown in Table 3. No statistically significant differences were noted between groups in terms of the rates of infiltration, occlusion, or phlebitis. The absolute risk reduction (ARR) was 6.07, which, as positive value, indicate that the CIPIR is an effective intervention and reduces the risk of adverse events. (Table 3).

## 5. Discussion

The results of this study showed that pediatric patients who received a CIPIR had a longer PVC indwelling time than those who received usual care. This result is similar to that of a study that indicated an average PVC indwelling time for a remaining catheter was 42.35 h, with 13% of pediatric patients having a PVC dwell time of >72 h, and 5.7% of pediatric patients having a PVC indwelling time of >96 h [10]. These findings suggest that CIPIR provides effective catheter coverage for pediatric patients and reduces invasive treatment.

The results of the present study showed that the rates of infiltration, occlusion, and phlebitis were not significantly different between the two groups studied. This finding is consistent with that of Garland et al. [7], who reported that replacing a catheter in critically ill children every 72 h would not decrease the incidence of phlebitis, bacterial colonization, or catheter-induced sepsis. These findings provide evidence to support the guidelines for the prevention of intravascular catheter-related infections [6], and suggest that avoiding the routine replacement of PVCs provides better patient care.

In this study, the average PVC dwell time was 113.06 h (SE = 2.42) for pediatric patients in the experimental group. The PVC dwell time of our subjects was longer than that reported in a study from a pediatric unit in Australia [10]. This difference may be due to a difference in the age groups studied. Most subjects in the Australian study were aged <1 year, but most of our subjects were aged 2–4 years. Close and continuous observation of PVCs for infection-related signs can prolong indwelling time and avoid the unnecessary invasive medical treatment of PVC replacement.

Although the results of the present study showed that CIPIR resulted in no statistically significant difference in the rate of phlebitis, the experimental group had a higher rate of phlebitis (5.7%) than the control group (1.4%). This is consistent with a review by Morrison et al. [20], in which they reported that replacing PVCs only when clinically indicated does not increase the patient risk of phlebitis or infection when compared to the current practice of routine replacement between 72 and 96 h in the adult patient population. These findings suggest that nurses should routinely assess the PVC insertion site and replace catheters only when signs indicate that this is medically necessary.

The results of this current study showed that the CIPIR was ineffective in improving the complications of PVC, this finding was inconsistent with previous studies that indicate that catheter-related infection did not increase during prolonged catheterization [14], that PVCs cause infiltration occlusion and phlebitis, and that the routine replacement of catheters is safe [11]. Differences between the results of the present and prior studies may be due to the subjects’ age, our study pediatric patients’ age were from 8 months to 12 years, and Bregenzer et al. [14] and Liu et al.’s [11] studies’ subjects were adult patients. Continued research to investigate whether age is a determinant of complications with PVCs is warranted.

Previous research has demonstrated that clinically indicated peripheral catheter replacement can decrease cost, discomfort to the patient, and nurses’ caring time [18]; thus, CIPIR saves cost, causes less pain, and requires shorter nursing care time. The current study did not involve these characteristics. Future studies are needed to ascertain the cost and patients’ perception related to the routine replacement of PVC or CIPIR.

This study has several limitations. First, the sample consisted only of pediatric patients from a single medical center in northern Taiwan, and may not fully account for the effects of differences in different age groups. Future studies may expand subject recruitment to different age groups in order to compare the effect of age variation on PVC indwelling time and complication rates. Second, we evaluated the effects of CIPIR on PVC indwelling time and the rates of complications using objective assessment, and the lack of subjective assessment may limit the generalizability of the results. Nevertheless, these results provide evidence that CIPIR is an effective alternative to usual care in pediatric patients. Thirdly, the size of the catheter and the type of drugs may influence the duration of the catheter, and this lack of involvement may limit the generalization of the results. Therefore, these variables should be used in future studies to more accurately and completely identify the size of the catheter and the type of drugs associated with PVC indwelling time and the rates of complications of PVCs. Fourth, the occlusion scale was self-developed, and possessed a lack of clinical metrics. Further testing and expansion of the use of this instrument for pediatric patients with other diagnoses is recommended. Finally, primary caregivers’ opinion regarding regular catheter replacement, which may have affected the clinically indicated peripheral intravenous replacement (CIPIR). Future studies are needed to determine the correlation between primary caregivers’ perspectives and CIPIR.

## 6. Conclusions

### 6.1. Conclusion

The use of CIPIR is effective in prolonging PVC indwelling time in pediatric patients with no significant increase in the rate of adverse events.

### 6.2. Clinical Implications

This intervention should be considered particularly because of the lack of a significant increase in the rates of infiltration, occlusion, or phlebitis between those receiving CIPIR and those receiving routine catheter replacement. In clinical care, healthcare providers should closely assess the complications of PVC, and provide adequate information to inform family caregivers’ of information regarding the timing of the replacement of PVCs.

## Figures and Tables

**Table 1:** Subject and disease characteristics by group (*n* = 283).

Characteristics	EG (*n* = 140)	CG (*n* = 143)	*χ^2^/t*	*p*
	*n*(%)/Mean (SE)	*n*(%)/Mean (SE)		
Age	3.44(0.34)	4.42(0.36)	1.939	0.053
Gender			1.849	0.174
Male	68(48.6)	81(56.6)		
Female	72(51.4)	62(43.4)		
Diagnosis			10.734	0.294 ^a^
Respiratory system	59(42.2)	76(53.1)		
Cardiovascular System	0(0)	1(0.7)		
Blood system	1(0.7)	1(0.7)		
Gastrointestinal system	24(17.2)	16(11.2)		
Urinary tract system	34(24.3)	28(19.6)		
Nervous system	1(0.7)	3(2.1)		
Skin system	10(7.1)	3(2.1)		
Musculoskeletal system	1(0.7)	1(0.7)		
Immune system	2(1.4)	3(2.1)		
Virus infection	8(5.7)	11(7.7)		
Catheter site			0.652	0.885
Right forearm or hand	60(42.9)	62(43.4)		
Left forearm or hand	64(45.7)	68(47.5)		
Right lower extremity	10(7.1)	7(4.9)		
Left lower extremity	6(4.3)	6(4.2)		
Hospital length of stay (LOS)	6.46(0.23)	5.88(0.24)		

^a^ Fisher’s exact test. EG =experimental group; CG =control group.

**Table 2:** Comparison of the indwelling time and complications of PVCs at post-test between the two groups (*n* = 283).

Characteristics	EG (*n* = 140)	CG (*n* = 143)	*χ^2^/t*	*p*
	*n*(%)/Mean (SE)	*n*(%)/Mean (SE)		
Indwelling time of PVCs (hour)	113.06(2.42)	65.67(0.88)	−18.447	0.001
Complication				
Infiltration			2.193	0.139
No	125(89.3)	119(83.2)		
Yes	15(10.7)	24(16.8)		
Occlusion			0.498	0.481
No	133(95.0)	133(93.0)		
Yes	7(5.0)	10(7.0)		
Phlebitis			3.865 ^a^	0.050
Level 0	132(94.3)	141(98.6)		
Level 1	8(100)	2(100)		
Level 2	0(0)	0(0)		
Level 3	0(0)	0(0)		
Level 4	0(0)	0(0)		

^a^ Fisher’s exact test. EG = experimental group; CG = control group.

**Table 3:** Relative risk, number needed to treat, relative risk reduction, and absolute risk reduction between the two groups (*n* = 283).

Characteristics	EG (*n* = 140)	CG (*n* = 143)	Odds Ratio	95%CI
	*n*(%)/Mean (SE)	*n*(%)/Mean (SE)		
Infiltration			0.595 ^a^	0.298–1.189
No	125(89.3)	119(83.2)		
Yes	15(10.7)	24(16.8)		
Occlusion			0.700 ^a^	0.259–1.894
No	133(95.0)	133(93.0)		
Yes	7(5.0)	10(7.0)		
Phlebitis			4.273 ^a^	0.891–20.488
Level 0	132(94.3)	141(98.6)		
Level 1	8(100)	2(100)		
Level 2	0(0)	0(0)		
Level 3	0(0)	0(0)		
Level 4	0(0)	0(0)		

^a^ 95% confidence interval for relative risk (RR). Number needed to treat = 16.47. Relative risk reduction, RRR = 0.168. Absolute risk reduction, ARR = 6.07.

## Data Availability

The data that support the findings of this study are available from the corresponding author. Restrictions apply to the availability of these data, which were used under license for this study. Data are available from the authors with the permission of Chang Gung Memorial Hospital Research Program in Taiwan.
